# Tissue Intrinsic Fluorescence Spectra-Based Digital Pathology of Liver Fibrosis by Marker-Controlled Segmentation

**DOI:** 10.3389/fmed.2018.00350

**Published:** 2018-12-11

**Authors:** Takashi Saitou, Sota Takanezawa, Hiroko Ninomiya, Takao Watanabe, Shin Yamamoto, Yoichi Hiasa, Takeshi Imamura

**Affiliations:** ^1^Department of Molecular Medicine for Pathogenesis, Graduate School of Medicine, Ehime University, Toon, Japan; ^2^Translational Research Center, Ehime University Hospital, Toon, Japan; ^3^Division of Bio-Imaging, Proteo-Science Center (PROS), Ehime University, Toon, Japan; ^4^Department of Gastroenterology and Metabiology, Graduate School of Medicine, Ehime University, Toon, Japan; ^5^Department of Lifestyle-related Medicine and Endocrinology, Graduate School of Medicine, Ehime University, Toon, Japan

**Keywords:** autofluorescence, spectral imaging, multi-photon microscopy, digital pathology, liver fibrosis, image segmentation

## Abstract

Tissue intrinsic emission fluorescence provides useful diagnostic information for various diseases. Because of its unique feature of spectral profiles depending on tissue types, spectroscopic imaging is a promising tool for accurate evaluation of endogenous fluorophores. However, due to difficulties in discriminating those sources, quantitative analysis remains challenging. In this study, we quantitatively investigated spectral-spatial features of multi-photon excitation fluorescence in normal and diseased livers. For morphometrics of multi-photon excitation spectra, we examined a marker-controlled segmentation approach and its application to liver fibrosis assessment by employing a mouse model of carbon tetrachloride (CCl_4_)-induced liver fibrosis. We formulated a procedure of internal marker selection where markers were chosen to reflect typical biochemical species in the liver, followed by image segmentation and local morphological feature extraction. Image segmentation enabled us to apply mathematical morphology analysis, and the local feature was applied to the automated classification test based on a machine learning framework, both demonstrating highly accurate classifications. Through the analyses, we showed that spectral imaging of native fluorescence from liver tissues have the capability of differentiating not only between normal and diseased, but also between progressive disease states. The proposed approach provides the basics of spectroscopy-based digital histopathology of chronic liver diseases, and can be applied to a range of diseases associated with autofluorescence alterations.

## Introduction

Cells and tissues of multicellular organisms intrinsically contain many kinds of chemical compounds that emit fluorescence. These include nicotinamide adenine dinucleotide (NADH), flavins, lipofuscin, melanin, porphyrins, collagen, elastin, vitamins, and other metabolites (1–4). To date, much of the attention about fluorescence microscopic analysis was on selective labeling with exogenous fluorophores or fluorescent proteins. In these types of analyses, native fluorescence was considered as background signals. However, these native molecules play important roles in tissue development, homeostasis, and disease progression, and thus can be used to reflect cellular states in living organisms. Therefore, monitoring the spatial distribution of endogenous fluorophores as biological markers in living systems enables us to explore cells and tissues in their original states.

Recent technological developments in fluorescence microscopy opened up a new window for use of endogenous fluorophores in clinical medicine. Multi-photon (MP) microscopy is a novel optical tool for intravital fluorescence imaging with high spatial resolution (5, 6). Due to its advantage of deeper tissue penetration, MP microscopy is suited for observation of *in vivo* molecular signals. Furthermore, second harmonic generation (SHG) enables direct imaging of molecules possessing non-centrosymmetric structures such as collagen and myosin (7). SHG and MP-based autofluorescence has been extensively used for the assessment of various diseases, including liver fibrosis and cancer (1, 2, 8–15).

Hyperspectral recording of intrinsic emission images make the label-free method especially informative (16–18). Native fluorescence in tissues generally shows broad spectral profiles, which are derived from a mixture of multiple distinct fluorophores. It is therefore difficult to separate individual sources of fluorescence due to their highly overlapping spectra. However, the spectral profile at each location in the images reflects a different biochemical composition. Thus, quantitative characterization of this spatial-spectral content of tissue intrinsic fluorescence would be useful for assessing tissue states. In microscopic analysis which uses targeted labeling with exogenous fluorophores, fluorescence source separation methods are commonly used. However, application of these methods to discriminate between the endogenous fluorophores is difficult, because of difficulty in obtaining the reference spectral data which reflect pure fluorophore sources. Therefore, in order to address this issue, instead of separating the distinct emission sources, in this study we took advantage of a different image processing technique called image segmentation.

The image segmentation approach is a powerful way for extracting spatial information from spectral images. Segmentation methods partition an image into non-overlapping homogeneous regions based on set criteria. A difficulty in hyperspectral segmentation is the construction of the appropriate criteria which separates spectra with several characteristic profiles. Some techniques have been applied for hyperspectral image segmentation, such as a watershed algorithm (19). Another approach proposed for image segmentation was internal marker-based segmentation. Markers are representatives of the spectra of typical objects in images, and hence are considered as bases for the spectra. Markers are often defined by flat zones, image extrema, or other morphological features (20–24). Once the markers are selected, it is easy to obtain accurate segmentation images by assigning pixels to the markers.

In this study, we aimed to apply this idea to the analysis of tissue intrinsic emission spectral images acquired using MP microscopy. We proposed a simple procedure for building internal spectral markers that reflects tissue-specific biochemical species, by combining a morphological feature extraction algorithm and a clustering method. We further developed an image processing pipeline for performing segmentation based morphometrics. This method was comprised of three successive steps—internal marker selection, image segmentation, and local morphological feature extraction. Image segmentation was performed using markers, which enabled us to apply mathematical morphology analysis. In order to extract the morphological features of the images, we employed an image patch-based approach, in which the local morphological feature of the spectra is represented as a collection of spectra within image patches. The local feature was applied to the automated classification test based on a machine learning framework.

In order to evaluate the feasibility of our method, we focused on liver fibrosis assessment. Liver tissue emits strong fluorescence, and tissue states associated with fibrosis have been analyzed using the SHG imaging technique (13, 25–31). These studies demonstrated that the SHG imaging of fibrillar collagen deposits correlates well with conventional scoring based on histological staining samples. Although information from native fluorophores is equally important as fibrillar collagen deposition, quantitative methods that utilize the full potential of MP-based fluorescence in early fibrosis assessment have been poorly investigated. Since autofluorescence can be used to evaluate cellular metabolic and inflammatory states, alterations in fluorescence could be potentially beneficial in detecting early fibrosis signals emanating prior to collagen deposition. In this study, we showed that the spectral imaging of native emission fluorescence from liver tissue has the capability of differentiating not only between normal and diseased tissue, but also between progressive disease states in early stages of fibrosis. The proposed approach provides the basics of imaging spectroscopy-based digital histopathology for diagnosis of chronic liver injury, and can be applied to a range of diseases associated with autofluorescence alterations.

## Results

### Intrinsic Emission Spectral Imaging of Mouse Liver Tissues by MP Microscopy

**Figure 1:**
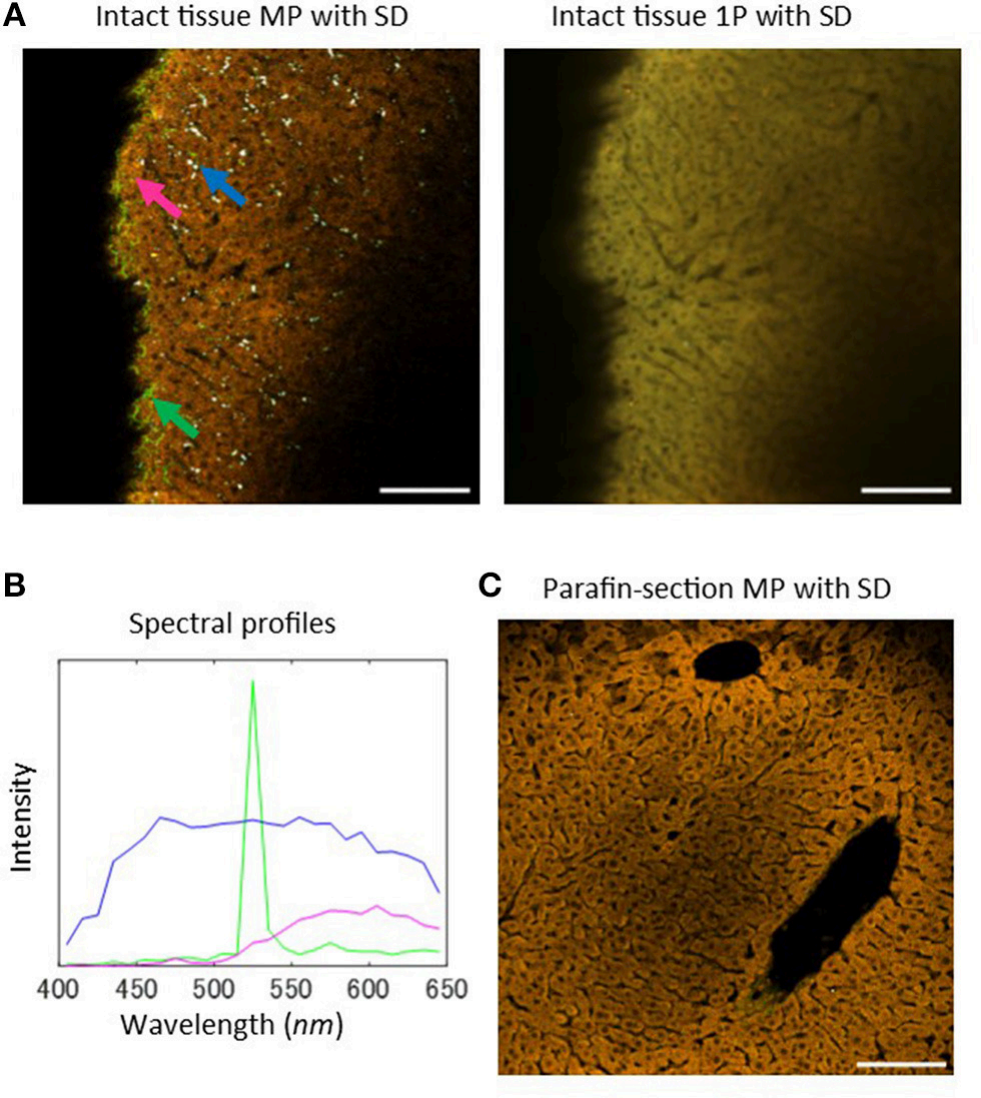
Intrinsic emission spectral imaging of healthy mouse liver tissues with MP microscopy. **(A)** Laser scanning microscopy images of intrinsic emission light for healthy and intact mouse liver tissues. Left: MP excitation image through the SD unit with an excitation wavelength of 1,050 nm. The emission spectra were detected as 25 channel images at a wavelength range of 400–650 nm with a bandwidth of 10 nm. Blue arrow indicates white-colored dot-like objects, green arrow indicates regions of emission of strong SHG signal, and magenta arrow indicates regions of hepatic parenchyma, primarily composed of hepatocytes. Right: 1P confocal laser scanning microscopy image through the SD unit. Four laser lines (405, 488, 561, and 640 nm) were used for excitation. **(B)** Profiles of the emission spectra of the indicated arrows in **(A)**. **(C)** Sectional examinations for liver tissues. MP excitation spectral image of a paraffin-section. Scale bar: 100 μm.

In order to investigate the usability of MP microscopy in evaluating liver tissue states, we began with a comparison of laser scanning microscopic images obtained from tissue intrinsic emission spectra including autofluorescence and SHG signals in healthy and intact liver tissue samples, without additional extrinsic marker fluorophores. The images were acquired by using a MP microscope through the spectral detector (SD) unit with a 1,050 nm wavelength excitation light (Figure 1A, left and Figure S1), and 1-photon (1P) confocal laser scanning microscope through the SD unit with four excitation laser lines with wavelengths of 405, 488, 561, and 640 nm (Figure 1A, right). In both images, hepatic parenchyma primarily composed of hepatocytes were captured in yellow to orange color signals, showing a regular array of hepatic cells. Vascular structures featured as dark regions, including sinusoids and portal and central veins, were also recognized in the images. The spectral profile of hepatic parenchyma marked by the magenta arrow in Figure 1A was plotted as a function of wavelength (Figure 1B, magenta line), indicating that the fluorescence intensity increased between 500 and 650 nm. This profile is consistent with the fact that NADH and flavins, which are major sources of intracellular fluorescence, have broader emission bands over 500 nm (1, 2, 32). The white dot-like structures possessing broader spectral profiles were observed only in the MP microscopy image (Figure 1A, blue arrow and Figure 1B, blue line), but not in the 1P microscopy image. These structures appeared to be concentrated on the periphery of the sinusoids. A possible source of this autofluorescence is retinol, a form of vitamin A. The SHG signal was detected at a wavelength of 525 nm, exactly half the wavelength of the excitation light, in the MP microscopy images, and highlighted the fibrous morphology of hepatic collagen fibrils. The signal showed sharp and narrow peak spectra (Figure 1B, green line). In the 1P microscopy image, only the hepatic parenchyma structures were recognizable, but not collagen fibrils or white dot-like structures. These data demonstrated a benefit of the use of MP microscopy.

Next, we performed analyses of MP excitation spectral imaging of tissue sections obtained from paraffin-embedded samples (Figure 1C). Hepatic morphology was well-characterized by their autofluorescence from hepatic parenchyma and SHG signals from collagen fibrils, but the white dot-like structures were not detected. This may be that retinol, a fat-soluble substance, disappears during the preparation of tissue sections. Therefore, these structures can be detected only in intact tissue samples, demonstrating a clear advantage of MP microscopy for evaluating intact liver tissue samples without the need for tissue section preparation.

### MP Excitation Spectral Imaging for Liver Tissues of CCl_4_-Induced Liver Fibrosis

**Figure 2:**
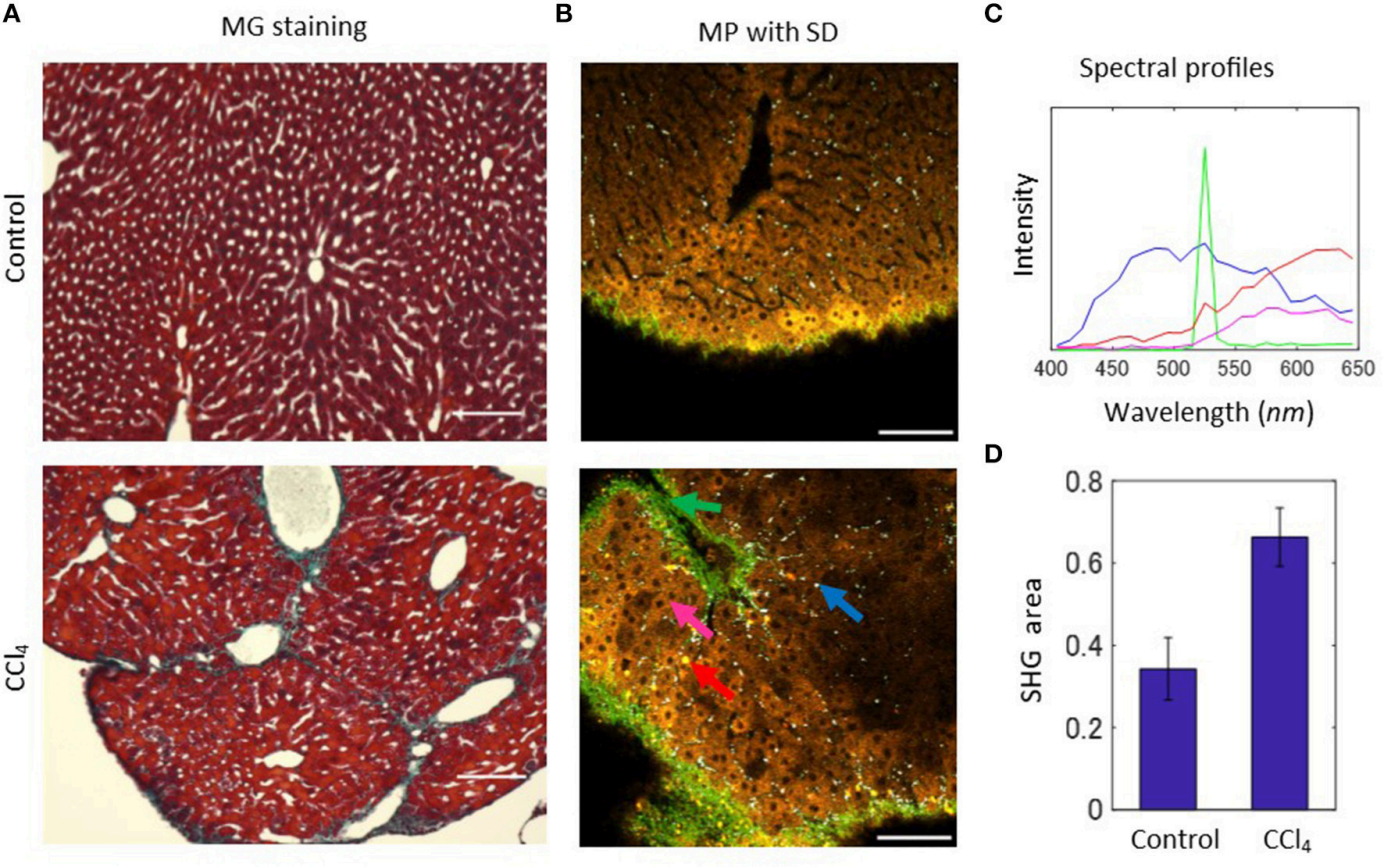
MP excitation spectral imaging for liver tissues of the CCl_4_-induced liver fibrosis model. **(A)** Histological images of the MG-staining sections for liver tissues of the control and CCl_4_ models. **(B)** MP excitation spectral images for liver tissues of the 2-weeks control and CCl_4_ model. In the CCl_4_ image, blue arrow indicates white-colored dot-like objects, green arrow indicates SHG, magenta arrow indicates hepatic parenchyma, and red arrow only observed in the CCl_4_ image indicates cellular-sized objects that appeared in yellow or orange color. **(C)** Profiles of the emission spectra of the indicated arrows in the control and CCl_4_ images in **(A)**. **(D)** Quantification result of the ratio of SHG signal area to the total image area. Scale bar: 100 μm.

In order to explore the feasibility of MP-based native emission spectra for the diagnosis of liver disease, we employed a mouse model of carbon tetrachloride (CCl_4_)-induced liver fibrosis. To focus on the detection of early signs of liver fibrosis, we generated the model mice by intraperitoneal administration of CCl_4_ twice a week for 2 weeks. The pathological states of the mice were investigated by a histological approach with the Masson-Goldner (MG) staining method, in which nuclei, cytoplasm, and connective tissues are stained in dark brown, red, and blue, respectively. Histological sections revealed that a representative image from the control groups shows normal hepatic cellular architecture (Figure 2A, upper), while that from the CCl_4_ group indicates an irregular array of hepatocytes, and an increase in collagen deposition around the portal vein (Figure 2A, lower). Excessive deposition of collagen forms bridges between portal veins. To evaluate these abnormal structures by intrinsic emission spectra, we acquired MP microscopy images of CCl_4_-exposed and control liver tissues. The images successfully captured areas of the extensive collection of collagen fibrils surrounding vessels and the Glisson's capsule—the surface layer of the liver—indicating fibrotic changes of collagen organization (Figure 2B). Furthermore, the accumulated collagens bridged between vessels, indicating consistency between MP microscopy images and histological images. In reflecting the fibrotic changes, the surface shape showed an undulated structure and depressed areas appeared in the images of the CCl_4_ model compared with those of the control model (Figure S2). Additionally, objects that emit a strong, orange-red colored fluorescence was observed (Figure 2B, red arrow). The size and form of these objects were similar to cells. The collection of spectra revealed that the intensity largely increases at wavelengths of 500–650 nm (Figure 2C, red line). This profile is similar to that from hepatocytes (Figure 2C, magenta line), but the fluorescence intensity of these red cell-like objects was higher than that of hepatocytes. These structures could be recognized in MP microscopy images of tissue sections. Comparison of the serial sections of the histological and MP microscopy images from paraffin-embedded samples revealed that the red cell-like objects exist where collagen is bridged between vessels (Figure S3). Therefore, these objects would be a sign of early fibrosis.

In order to estimate the rate of excessive collagen fibril accumulation, we quantified the SHG images extracted from the spectral images. The z-stack image sequences were converted to maximum intensity projection (MIP) images, in which the highest pixel intensity among the images in the z-stack was projected on to a new single-layer image (Figure S4). Then, these images were binarized with appropriate thresholding, before performing the calculation of the ratio of the SHG area to the total image area for both the CCl_4_-exposed and control samples (Figure 2D). The results showed increased areas of collagen fibers in the CCl_4_ model compared with control samples, quantitatively confirming the excessive collagen deposition.

### Selection of Internal Markers

**Figure 3:**
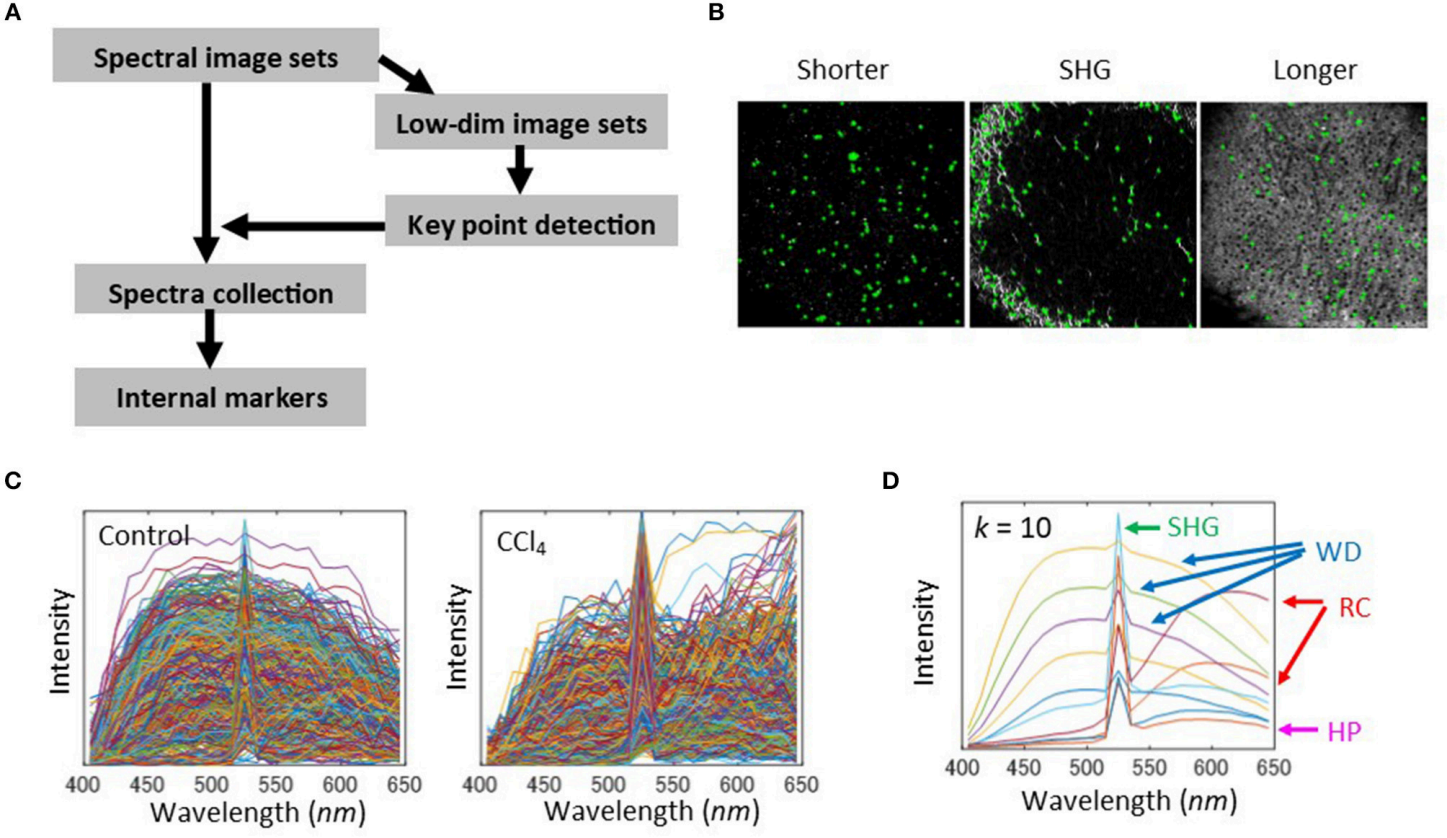
Selection of internal markers. **(A)** Schematic of the image processing workflow. First, the dimensionality of spectra was reduced by defining color bands of shorter wavelengths 400–500 nm, SHG 520–530 nm, and the longer wavelengths 550–650 nm. The lower dimensional images were generated by averaging the spectra intensities among each of the bands. Using these image sets, a feature detection algorithm was applied to detect key points in the images. With the coordinates of the detected points, spectra were collected from the original spectral image sets and were stored in a spectral pool. Next, the pooled spectra were quantized to the centroid values of a *k*-means cluster, in which each cluster represents a spectral marker. **(B)** Key point extraction was by FAST feature detection algorithm. The results for bandpass images with shorter wavelengths, SHG, and longer wavelengths were shown. The detected points were denoted as green cross marks. **(C)** Spectral profiles collected from the detected key points for the 2-weeks control and CCl_4_ model. **(D)** Quantization of the collected spectra for the cluster number *k* = 10 of *k*-means clustering. Interpretation of quantized spectra is indicated for HP, SHG, WD, and RC.

As seen previously, native fluorescence in liver tissue showed broad spectral profiles. It is therefore difficult to discriminate individual sources of fluorescence, and this problem is associated with the limited availability of reference data. However, the spectral profile at each location in the images corresponded to the liver disease state. Thus, for quantitative evaluation of liver tissues, we employed an image segmentation approach, called marker-controlled segmentation (23, 24), instead of attempting to separate the various sources of fluorophores. The basic idea of this approach is to find internal markers, which indicate significant objects appearing in spectral images. Possible markers need to include manually selected spectra (Figures 1B, **2C**). Hence, in order to examine the reproducibility of these spectral profiles using automated algorithms, we first attempted to construct markers by performing a pixel-by-pixel collection of spectra and classifying the spectra using the *k*-means clustering method. The spectral profiles that were obtained include ones which were similar to manually selected ones, a sharp peak profile of SHG, broader profiles of white dots (WD), and hepatic parenchyma (HP) and red cell (RC)-like structures at the longer wavelength with low and high intensity levels, respectively (Figure S5). However, this method of calculation requires high computational costs because of the large size of the collected spectra. Therefore, we looked for an alternative way of constructing markers. For computational cost-savings, we used dimensionality reduction and morphological feature extraction techniques. This workflow is shown in Figure 3A. First, to make the high dimensional images easy to handle with general image processing techniques, we performed a dimensionality reduction of spectral images to three image types. These were defined by wavelengths: 550–650 nm (longer wavelength band), 520–530 nm (SHG band), and 400–500 nm (shorter wavelength band). Next, we ran a morphological feature detection algorithm, called features from accelerated segment test (FAST)-algorithm (33), to collect key points of these three types of images independently (Figure 3B). At each point, the spectral profile was collected for the control and CCl_4_ models (Figure 3C). Finally, the spectral markers were created by quantizing these collected spectra. This was performed by the *k*-means clustering method (Figure 3D). In the case of the clustering number *k* = 10, the obtained spectral profiles resembled those that were obtained by pixel-by-pixel construction. The spectral curve can be interpreted as noted. The *k* = 20 case can also characterize the typical features of the spectra, while in the *k* = 4 case, the peak intensity of the profile reflecting the SHG signal (Figure S6, blue line) was not as high as that of the SHG profile in the *k* = 10 case.

### Marker-Controlled Image Segmentation and Mathematical Morphology Analyses

**Figure 4:**
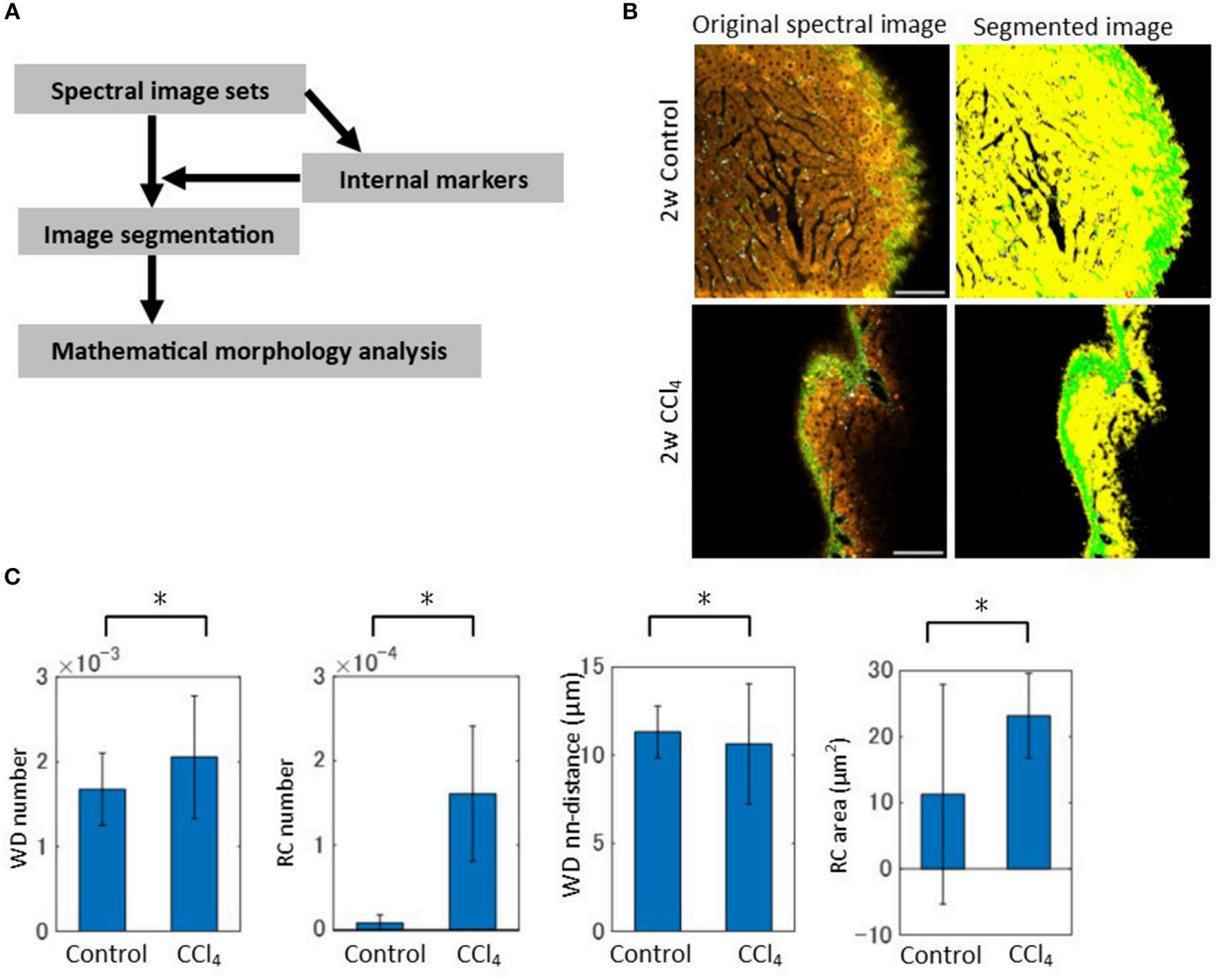
Image segmentation and mathematical morphology analyses of spectral image data. **(A)** Schematic of the image processing workflow. The segmentation of spectral images was performed by assigning each pixel to the nearest centroid of the markers. The mathematical morphology analysis was then performed on the segmented images. **(B)** Original and segmented images reflecting the regions of HP, SHG, WD, and RC are shown. Segmented images representing HP, SHG, WD, and RC are in yellow, green, white, and red, respectively. **(C)** Mathematical morphology analyses of the segmented images. The graphs shown are the ratios of the number of WD sites and RC sites to the total signal area, the averaged nearest neighbor distance between WD sites, and the averaged areas of RC to the total signal area. Asterisks indicated statistical significance with the Kolmogorov-Smirnov test with a *p* < 0.05.

Image segmentation was performed in such a way that a spectrum at each pixel was assigned to the nearest centroid of the constructed spectral markers (Figure 4A). Thus, each pixel was *k*-valued, except for low signal pixels, which were excluded in the analysis. To confirm that the image segmentation method worked well, we compared SHG images between the spectral band (520–530 nm) extracted images and marker-controlled segmentation images from the spectral and three-channel image data obtained through the emission filter sets. For image segmentation of the emission filter-based images, we followed the same procedure used in the analysis of the spectral images. The ratios of overlapping pixels to the total signal area with respect to the SHG signal of the *k* = 10 case between the spectral-band segmentation images and the marker-controlled segmentation image sets were calculated. The results for spectral data demonstrated that over 98% of the regions coincided (Figure S7). On the other hand, the ratios calculated from the images through the emission filters showed that <90% of the regions coincided. Furthermore, the segmented images from the spectral data separated the individual objects well (Figure S8), while the segmented images from the emission filters seemed to differentiate the objects less (Figure S9). These data demonstrated that there was an improvement in segmentation performance for images when spectral detection was used, rather than emission filter-based multi-channel detection. The segmented images can be interpreted as SHG, WD, HP, and RC objects by the spectral marker profiles (Figure 4B and Figure S8). These image data made it possible to perform morphometric analyses using mathematical morphology. We quantified the ratios of the number of WD and RC sites to the total signal area. These data revealed increased numbers of objects in the CCl_4_ model compared with the control model (Figure 4C). To further explore the morphological characteristics of the images, local density of the WD sites and averaged areas of the RC objects were calculated (Figure 4C). The local density of the WD sites measured by the nearest neighbor distances between the objects demonstrated that local accumulation of the sites are enhanced, and the areas of RC objects dramatically increased, in the CCl_4_ model. Therefore, these morphometric analyses successfully characterized the pathophysiological image patterns of the WD and RC objects in liver tissues.

### Local Morphological Feature Description and Automated Image Classification Test

**Figure 5:**
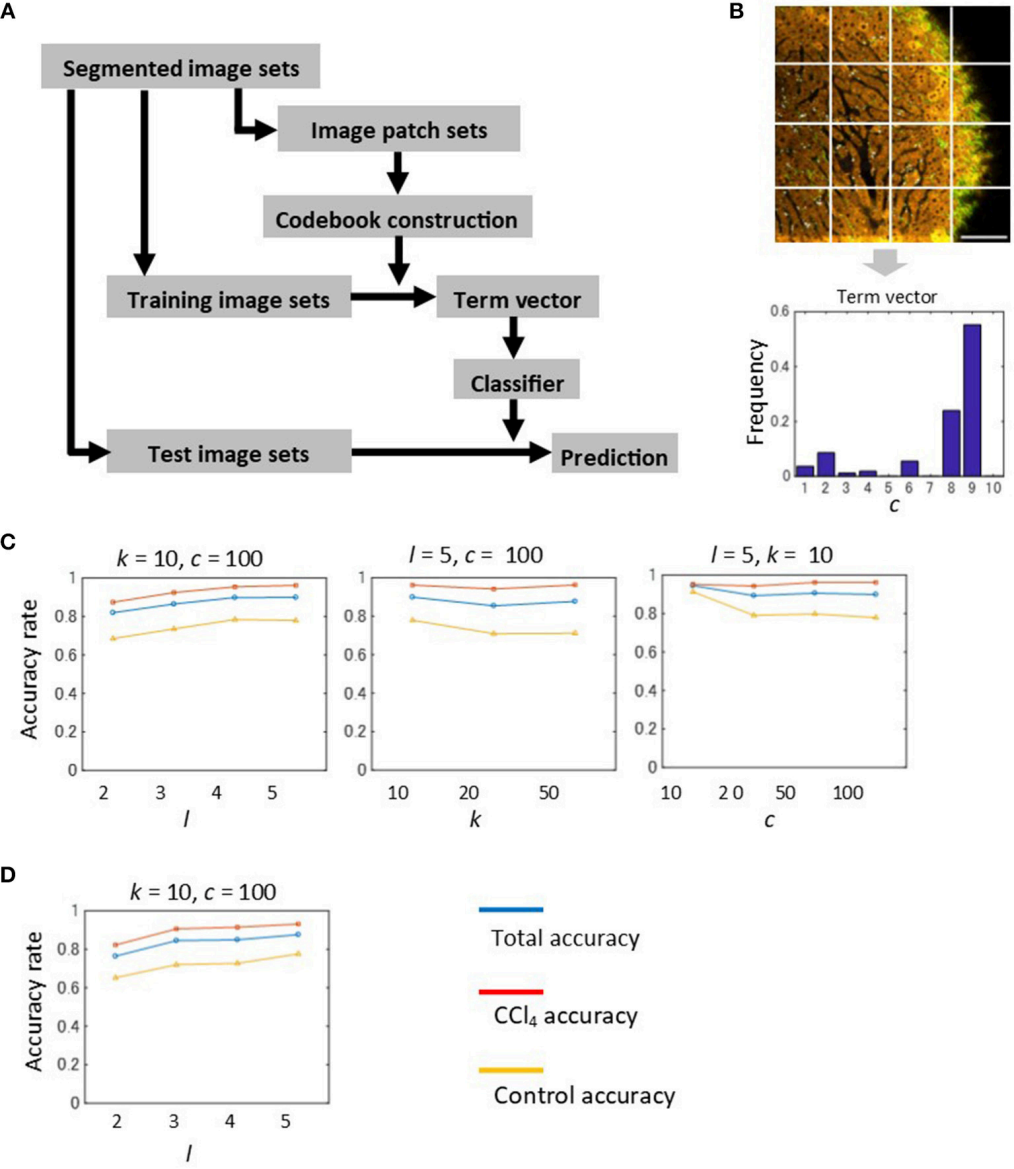
Automated image classification test by the BoF machine learning method. **(A)** Procedure of the BoF framework. BoF is comprised of three steps, codebook construction, training, and testing. In the codebook construction step, image patch sets were created and feature vectors corresponding to the image patches were calculated. The feature vectors were defined as histograms of spectral markers collected within image patches. Visual words, which comprise the codebook, were created from these vectors by performing vector quantization. In the training step, an image was represented as a set of image patches that are subjected to feature vector calculations and visual word assignments. A histogram of visual word occurrences (term vector) was subsequently calculated for each image. Based on these data, the SVM classifier was constructed. In the test step, image prediction was performed based on the classifier. **(B)** Term vector calculation for an image. A divided image using the regular square grid with the division level *l* = 2 was shown. An image which is represented as a set of visual words was converted to a term vector. **(C)** Results of the classification test performed on 2-weeks models. The machine learning parameters are the division level *l*, the cluster number of the spectral markers *k*, and the codebook size *c*. The total, CCl_4_, and control accuracy rates are depicted as blue, red, and yellow lines, respectively. **(D)** Results of the classification test performed on 2-weeks models excluding SHG marker information. The total, CCl_4_, and control accuracy rates are depicted as blue, red, and yellow lines, respectively.

So far we showed that the morphological features of individual segmented objects could characterize liver tissue states. This suggests that local information of quantized spectra can be used as image features for image recognition. Thus, it is worth investigating whether simple construction of feature vectors, such as local sets of spectra, would be advantageous in automated image classification. In order to investigate this point, we employed a machine learning method for image classification, called the Bag-of-Features (BoF) framework (34–36). This method is described briefly in Figure 5A. A key to this algorithm is to construct the codebook comprising a set of quantized vectors, called visual words, which describe image patterns of image patches. Image patches were created using a regular square grid, which divides images with a division level *l* (Figure S10). We represented a feature vector of an image patch as the frequency of spectral markers (Figure 5B). Then, the feature vectors of the image patch sets were used to create visual words by performing vector quantization of size *c*. In the training step, the term vector, a histogram of visual word occurrences for each image, was calculated based on visual words. The BoF algorithm depends on three parameters, namely the number of markers *k*, the image division level *l*, and the codebook size *c*. We investigated parameter sensitivities for image classification. We evaluated classification performance by overall accuracy and individual accuracy for the control and CCl_4_ model at 2 weeks. The results were shown as averaged values performed on *k* = 4-fold cross-validation test. The numbers of images used for training and test samples are summarized in Figure S11. Overall, the highest classification performance achieved over 95% accuracy. The accuracy of the CCl_4_ model was higher than that of the control model. The performance depended on the division level, i.e., the accuracy increased as the division level increased (Figure 5C and Figure S12A). This indicated that the classification became more accurate as the image patch size gets smaller and division level number becomes larger. The number of markers did not affect the results greatly (Figure 5C, flrefsup1Figure S12B), suggesting that there is no need to create a larger number of markers. We observed no clear advantage for using a larger codebook size (Figure 5C and Figure S12C). When the analysis was applied to the images which excluded the SHG markers, the accuracy showed a level equal to that of full markers, indicating that the liver pathological state in CCl_4_-induced liver injury can be classified without the use of collagenous fibrillar information (Figure 5D).

### Spectral Morphometric Analysis: 2- vs. 4-Weeks CCl_4_-Exposed Model

**Figure 6:**
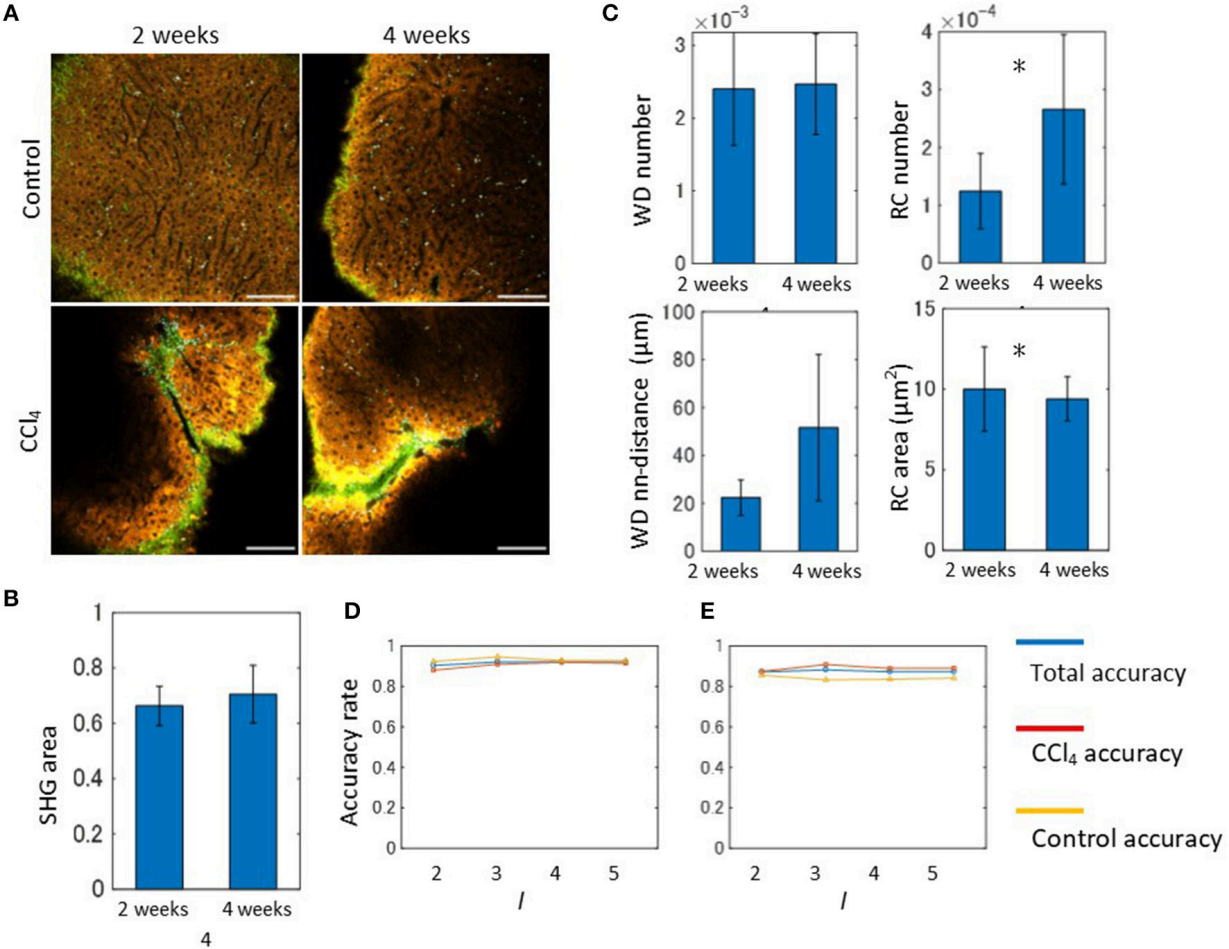
Spectral image morphometry: 2-weeks model vs. 4-weeks model. **(A)** MP excitation spectral imaging for liver tissues of the 2- and 4-weeks CCl_4_ models. Scale bar, 100 μm. **(B)** Quantification result of the ratios of SHG signal area to the total image area. **(C)** Mathematical morphology analyses performed on the 2- and 4-weeks CCl_4_ model. The graphs shown are the ratios of the number of WD sites and RC sites to the total signal area, and the averaged nearest neighbor distance between WD sites, and the averaged areas of RC to the total signal area. Asterisks indicate statistical significance with the Kolmogorov-Smirnov test with *p* < 0.05. **(D)** Results of the automated classification test performed on the 2- and 4-weeks CCl_4_ models. The total, CCl_4_, and control accuracy rates are depicted as blue, red, and yellow lines, respectively. **(E)** Results of the classification test performed on the 2- and-4 weeks CCl_4_ models excluding SHG marker information. The total, CCl_4_, and control accuracy rates are depicted as blue, red, and yellow lines, respectively.

In order to apply our algorithm to the discrimination of the liver fibrosis pathological state, we used the 2- and 4-weeks CCl_4_-exposed mouse models. Representative emission spectral images of these models are shown in Figure 6A. In contrast to the control models, images of CCl_4_ models exhibited abnormal signal patterns. As observed in the 2-weeks CCl_4_ model, the undulated liver surface with extensive collagen fibrosis and RC objects were also observed in the 4-weeks model. Although these observations reflected typical features of hepatic pathology during fibrosis progression, it is difficult to distinguish the two model images without quantitative image analysis. For the purpose of quantitative evaluation of hepatic morphology associated with fibrosis, we performed image morphometric analysis as was done for the 2-weeks model previously. First, the ratios of the SHG area to the image total area for both the 2- and 4-weeks CCl_4_-exposed models were calculated using the MIP method as was done previously (Figure 6B). The results showed that the collagen fibril deposition on the surface area of the liver did not change. However, this did not indicate that the fibrosis would not proceed, as the estimation of the ratios of the SHG area in internal areas using tissue sections revealed an increased collagen amount in the 4-week model when compared to the 2-week model (Figure S13). To proceed further, we performed image segmentation of the spectral images with the marker size *k* = 10. The mathematical morphology analyses revealed that the ratios44 of the number of the WD sites to the total area indicated no significant differences between the models, while those of the number of RC sites becomes larger as the period of the drug administration becomes longer (Figure 6C). The local density of the WD sites indicated no significant differences. The averaged areas of the RC increased in the 4-weeks CCl_4_ model compared to those of the 2-weeks model. Therefore, only the image patterns of the RC objects characterized the liver tissue pathological state between the 2- and 4-weeks models. We next performed an automated classification test using the BoF framework. The numbers of images used for training and test samples are summarized in Figure S11. Overall, the prediction accuracy achieved ~90% accuracy (Figure 6D). Possibly because of its very high accuracy rate, the result did not show any division level dependence. Application of the analysis to images excluding the SHG information provided an equal level of accuracy to that achieved from full markers (Figure 6E).

## Discussion

In order to establish a digital histopathological assessment for liver fibrosis, we used liver tissue intrinsic emission spectra-based analysis, and demonstrated a methodology for performing image morphometric analysis. This method was composed of three successive tasks: selection of internal spectral markers, image segmentation followed by mathematical morphology analysis, and construction of spectral feature vectors followed by an automated classification test with a machine learning method. We evaluated the potential utility of this method for classifying images derived from liver tissues as either normal or diseased based on the mouse model of CCl_4_-induced liver fibrosis. We showed that our method possesses high classification performance not only between normal and diseased states, but also between progressive disease states. Therefore, the proposed image processing pipeline would be a promising platform of spectral morphometrics for tissue fluorescence diagnosis of various tissues or organs.

### MP Excitation Intrinsic Emission Spectra in Liver Tissue

MP microscopy is becoming a promising tool for observing living, thick, and opaque tissues in a non-invasive manner. Label-free imaging using this method enables us to observe unstained samples using endogenous sources of nonlinear signals and to diagnose several types of disorders. In the liver, fibrosis assessment based on the SHG signal, which comes from collagen molecules upon two-photon excitation, has been extensively investigated (13, 25–31). Liver fibrosis is characterized by the excessive accumulation of collagen in the extracellular matrices of the liver tissue (37). Changes in collagen architecture that occur in the liver can be obtained by this high resolution imaging method. Therefore, SHG has been used to quantitatively characterize fibrillar collagen deposition, which was shown to correlate with conventional histological analyses. On the other hand, strong native fluorescence is also emitted in liver tissue. Endogenous fluorescent molecules found in tissues include NADH, flavins, lipofuscin, porphyrins, and vitamins (1–3). Fibrosis progression is related to cellular, fibrotic, and microvascular changes, therefore monitoring the histopathological information connected with liver fibrosis is key to the diagnosis of chronic liver diseases. In our microscopy settings that use an MP excitation laser at a 1,050 nm wavelength, the primary intracellular sources of fluorescence in liver tissues are NADH, flavins, and retinol, with absorption maxima of 350–400 nm and emission at 400–600 nm. In contrast, for tryptophan and its indoleamine derivatives, the absorption maxima is at a wavelength of <300 nm and emission at a wavelength <400 nm. NADH and flavins allowed us to visualize silhouettes of hepatic cells and to discuss histological characteristics. In the CCl_4_-admistered liver tissue samples, we observed strong fluorescence at a longer wavelength range of 500–650 nm, which was possibly derived from lipofuscin or porphyrin. These have been reported to emit longer wavelength fluorescence and are associated with inflammation or cell damage (1–3). Thus, this object is a possible sign of early fibrosis progression. However, since this type of structure could not be detected in histological sections, and its origin is still not clear, further studies on the functional roles of this fluorescence in liver injury are required. Vitamin A, with a maximum excitation wavelength of 350 nm and a broad emission spectrum of 400–600 nm (38), was detected as white dot-like objects across the sinusoids. This suggested that retinol is excited with the three-photon absorption process around the 350 nm. It is well-known that this vitamin plays an important role in liver homeostasis. Hepatic stellate cells store vitamin A and their activation is related to hepatitis. Hepatic stellate cells are considered to be the principal effector of liver fibrogenesis, and are localized at the space of Disse, located between the sinusoids and the hepatocytes. Thus, these morphological features are consistent with the fact that the white dot-like structures are primarily derived from vitamin A fluorescence. Moreover, we confirmed the specific distributions of stellate cells around vessels in CCl_4_ model tissues using immunohistochemical staining with α-SMA (Figure S14). Additionally, we found that the vitamin A signal disappeared in paraffin sections. Therefore, intact tissue observation is well-fitted to MP microscopy and the information gathered regarding the fluorophores excited at ultraviolet wavelengths shows its merit.

For further investigations on whether variation in MP excitation wavelength alters image features, we performed MP microscopy imaging through emission filters with three different excitation wavelengths at 800, 1,050, and 1,200 nm (Figure S15). Autofluorescence from hepatic parenchyma and the white dot-like structures (detected in the 1,050 nm excitation image) was also observed in the 820 and 1,200 nm excitation images, although the efficiency of the excitation seems to decrease in the 1,200 nm excitation images. The broad excitation wavelength of this object indicates flexibility of wavelength adjustment, suggesting the possibility for simultaneous imaging of hepatic stellate cells with exogenous fluorescent markers such as GFP.

### Marker-Controlled Image Segmentation Approach for Spectral Morphometry

In the labeling of fluorescence such as fluorescent proteins and fluorescence emitting chemical compounds, source spectral profiles can be measured and these can be used in source separation methods. However, the intrinsic emission spectra are composed of a mixture of several fluorophores and hence showed broad spectral profiles, making it difficult to separate individual sources of fluorophores. For morphometric analysis of spectral images, in this study, we considered an image segmentation approach in which bases of spectra, called internal markers, were chosen from the original images. The idea behind this is that the spectral profile at each location in the images reflected a different biochemical composition and thus spatial-spectral information of liver tissue intrinsic fluorescence has the ability to describe liver tissue states. In order to select markers, we employed a combination of dimensionality reduction, a feature detection scheme, and clustering of spectra. The reason for doing this was to reduce computational cost, and to simplify the process for obtaining markers as much as possible. Compared with the manually selected spectral profiles, we showed that the profiles reproduce the ones typical of those corresponding to significant objects in the images: a sharp peak profile of SHG, broader profiles of white dots, and hepatic parenchyma and red-cell like structures at the longer wavelength and low and high intensity levels, respectively. Different techniques for spectral marker selection were proposed based on spatial features such as flat zones or image extrema, and these techniques were applied in the field of general scene recognition and geoscience (20–23). Furthermore, image patch-based feature descriptors which use the Gabor filter were proposed to include spatial and spectral information (39). Development of a marker selection scheme by incorporating these spectral-spatial feature extraction methods may improve the segmentation results, and is a subject of future research.

### Classification Performance of the BoF Machine Learning Method

The discrimination ability of the spectral imaging data was evaluated using an automated classification test by the BoF machine learning framework. For this, we defined a local feature vector by a histogram of spectral markers within a local patch. The highest accuracy we obtained was over 95% for the 2-weeks control and CCl_4_ model, as well as for the 2- and 4-weeks CCl_4_ model. We investigated how parameters influence the results. The parameters we have are the division level *l*, the number of spectral markers *k*, and the codebook size *c*. As the division level, which is equal to the number of patches per image, increases so does the classification accuracy. This means that the higher accuracy requires use of a large number of image patches, resulting in higher computational demand. On the other hand, the number of spectral markers did not affect the results, and the improvements on the increased codebook size was not clearly observed. These data indicated that no greater values for the marker number and the codebook size are required, suggesting low computational cost. When information about SHG signal markers was excluded in the classification analysis, the accuracy did not change compared with that performed on full spectra. This indicated that the liver pathological state in CCl_4_-induced liver injury can be classified only through use of autofluorescence. The mathematical morphology analyses also supported this result, i.e., differences between the CCl_4_-induced liver injury models were only recognized in the image patterns of RC autofluorescence. This provides a potential application of our methods to the diagnosis of diseases that are associated with autofluorescence alteration, but do not yet show fibrotic changes. Therefore, it would be interesting to investigate acute liver injury models using our method.

### Digital Diagnosis of Liver Fibrosis

Liver biopsy is the gold standard for staging liver fibrosis progression although it has the risks of sampling error and of being an invasive procedure. Grading and scoring methods using stained liver tissue sections have been developed (40–43). However, these methods are descriptive or semi-quantitative and the results therefore suffer from inter/intra observer discrepancies (44, 45). Although an automated quantification algorithm for fibrosis evaluation have been developed (46, 47), inter-assay differences such as staining variability, which biases reproducibility and objectivity of the quantification method, still remain. Therefore, stain-free imaging methods using MP and SHG microscopy combined with automated quantification methods have been developed for liver fibrosis assessment. The feasibility of these methods for clinical diagnosis was validated using biopsy specimens from human patients (25, 27, 28, 48). While extensive investigations on SHG-based fibrillary collagen assessment were made, fluorescence information from cells and tissues in MP microscopy images remains poorly exploited. We here showed that autofluorescence observation with quantitative morphometric analysis allowed us to discriminate liver disease not only between normal and CCl_4_-induced diseased states, but along the progression of liver fibrosis. Although the usability of our method was shown only in the drug-induced liver fibrosis models, our quantification methodology using intrinsic emission spectra and a computational pipeline possibly contributes as a multifaceted evaluation of chronic liver diseases, such as nonalcoholic steatohepatitis (NASH) or hepatocellular carcinoma (HCC).

Development of endoscopic techniques using MP microscopy is of great importance for the clinical application of MP microscopy, which allows real-time histological examination of the liver without conventional biopsy for liver disease diagnosis. A miniaturized probe for MP microscopy were developed (49, 50), together with the development of a quantitative technique for analysis of data, which may be advantageous for overcoming constraints of nonlinear microscopy in clinical studies.

## Materials and Methods

### Ethics Statement

This study was carried out in accordance with the recommendations of the Guidelines for Animal Experiments of Ehime University, the Ethics Committee for Animal Experiments of Ehime University. The protocol was approved by the Ethics Committee for Animal Experiments of Ehime University.

### Mouse Model of Carbon Tetrachloride (CCl_4_)-Induced Liver Fibrosis

Male C57BL/6J mice at 5 weeks of age were purchased (CLEA Japan, Inc.), and these mice were divided into four groups, 2-weeks CCl_4_ (*n* = 3), 4-weeks CCl_4_ (*n* = 3), 2-weeks control (*n* = 2), and 4-weeks control (*n* = 3). To produce mouse models of CCl_4_-induced chronic liver injury in which prolonged administration leads to liver fibrosis, cirrhosis, and hepatocellular carcinoma (51), intraperitoneal injection of 20% CCl_4_ dissolved in olive oil was administered twice a week at a dose of 0.2 mL/100 g body weight for 2 weeks (2-weeks CCl_4_ group) and 4 weeks (4-weeks CCl_4_ group). For control groups (2-weeks control and 4-weeks control), olive oil was administered. The mice were sacrificed via cervical dislocation, and then cardiac perfusion with phosphate-buffered saline (PBS) was performed to flush out blood, before collection of liver tissues. The harvested liver samples were fixed overnight at 4°C in 4% paraformaldehyde (PFA) in PBS.

### Preparation of Tissue Sections

After fixation of liver tissues, samples were soaked in PBS for a least a day prior to analyses of the tissue sections. For preparation of the tissue sections, the samples were embedded in paraffin. The paraffin-embedded samples were cut into 5 μm thick sections. The sliced sections were then deparaffinized with xylene, and subjected to microscopy. The sections subjected to the histological analysis were stained with the Masson-Goldner staining technique. Bright field images of the sections were acquired using a wide field inverted microscope (All-in-one fluorescence microscope BZ-X700, Keyence, Inc.) with a 20 × magnification objective lens (PlanFluor 20 × NA:0.45, Nikon). Image acquisition of the sections using confocal laser scanning and a multi-photon excitation microscope are described below.

### Image Acquisition by Laser Scanning Microscopy

In order to perform microscopic image acquisition, we utilized an upright confocal laser scanning (A1R, Nikon, Inc.) and multi-photon (MP) microscope (A1R-MP, Nikon, Inc.). The microscopes were equipped with a water immersion objective lens (CFI75 Apo 25 × W MP, NA:1.1, Nikon, Inc.), and the non-descanned detector (NDD) unit and the spectral detector (SD) unit (A1-DUS, Nikon, Inc.). Emission spectra were detected at a wavelength range of 400–650 nm with a bandwidth of 10 nm (recorded as 25 channels in total), through the SD unit. A Ti:sapphire laser oscillator (Insight DeepSee, Spectra-Physics, Inc.) and four confocal laser lines (405, 488, 561, and 640 nm) were used for excitation. For the detection of MP excitation fluorescence and SHG signals, excitation wavelengths of 800 nm, 1,050 nm, and 1,200 nm were used. MP excitation spectral images were acquired at a wavelength of 1,050 nm. Emission filter sets used for detection through the NDD unit in MP microscopy were as follows: (1) the dichroic mirror (DM) 450 nm and the bandpass filter (BF) 417/60 nm (center wavelength/bandwidth), (2) DM 560 nm and BF 525/50 nm, (3) DM 662 nm and BF 617/73 nm. The field of view of the acquired images was 0.5 mm × 0.5 mm and the resolution was 512 × 512 pixels. The images originally recorded as 12-bit gray level images were converted to 8-bit gray level images when analyzed computationally. To observe intact hepatic tissues, excised tissues (middle lobe of the liver) were embedded in 1% agarose in a plastic dish and the liver mid-ventral aspect facing upwards was exposed under the objective lens of the microscopy system as previously described (13). The images were acquired as *z*-stack image sequences with a step size of 2 μm ranging from the deepest portions (100~200 μm in depth) to the surface of liver tissue. In total, 3–4 regions for each mouse were imaged. To observe tissue sections, sections mounted on glass slides were placed on the microscope stage before acquiring images.

### Image Processing

#### Internal Marker Selection

The process of internal marker selection consisted of four parts: (1) dimensionality reduction of spectral images, (2) key point detection by a feature detection algorithm, (3) spectra collection, (4) spectra quantization. First, dimensionality of spectra was reduced through a projection of a space of spectrum to a space with few dimensions. We simply defined three color bands, the shorter wavelength band 400–500 nm, the SHG band 520–530 nm, and the longer wavelength band 550–650 nm, because this is similar to the RGB color space, which is the simplest choice for manipulating color. Then, the three band images were created by integrating spectral signal intensities within the defined bands. After the conversion to images with low dimensionality, which in turn can be easily handled with general image processing methods, features from the accelerated segment test (FAST)-algorithm (33) were applied for each image to detect spatially localized features, such as corners. Next, from the detected feature points, the spectra were collected from the original spectral image sets, and were stored in a pool of spectra. When collecting spectra, in order to reduce signal noise, we used the structure elements of disk with a 2 pixel radius and averaged the collected spectral signals within this region. Finally, we created markers of spectral vectors by performing quantization of the collected spectra. In order to do this, we used a *k*-means clustering method with *k* = 4–20. All the extracted spectra are thereby partitioned into *k* regions in which each spectral vector belongs to the region with the nearest centroid.

#### Image Segmentation and Mathematical Morphology Analyses

The segmentation of spectral images was performed using the spectral markers. A spectral vector collected from each pixel was assigned to the nearest centroid of the markers. *k*-valued images were created by doing this for every pixel of the images. For spectrum assignment, we selected high signal pixels in which the intensities averaged over spectral bands are larger than 4, to avoid the inclusion of low signal pixels in the following image analysis. The mathematical morphology analysis was applied to the segmented images. This includes extracting regional properties of simply-connected objects, such as number and areas of the objects, and distances between the objects. The total signal area in an image was defined as the sum of the high signal pixels as defined above.

#### Automated Image Classification Using the BoF Machine Learning Method

We employed the BoF framework for an automated image classification strategy, as previously described (36). This method has been applied to the classification of two photon excitation fluorescence images (35, 52). This approach to image classification is based on an unordered collection of image feature descriptors derived from local patches. Thus, the image was represented as a histogram of the number of occurrences of particular patterns in a given image, and the histograms were subjected to a machine learning based classification test.

In order to obtain local feature descriptors, we first created the image patch sets. We used a regular square grid for image division to represent images as a collection of image patches to be subjected to feature extraction. The division levels employed were *l* = 2, 3, 4, and 5, hence each extracted image patch size was 128 × 128, 64 × 64, 32 × 32, and 16 × 16 pixels, respectively. We represented a feature vector of an image patch as a histogram of the spectral markers. This calculation was performed individually on each image patch. The next step was to construct the codebook which consisted of quantized vectors in a feature space called visual words. The feature vectors were extracted from the image patches from each image and added to the codebook feature space. To develop the codebook, *k*-means clustering with size *c* = 10, 20, 50, 100 was performed. The feature vectors were thus quantized to centroid values in a codebook space, in which each cluster represented a visual word. Within the BoF framework, an image was represented as term vectors, a histogram of visual words. Given an image in the sets, features were detected and assigned to the nearest codes in the codebook. We employed a *k*-nearest neighbor (kNN) classifier to assign the extracted images to the closest terms in the codebook. For machine learning, we used the SVM classifier. The BoF parameters we used were the division level of the image, *l* and codebook size, *c*. The division level controlled image patch size and sampling density. The codebook size, which corresponded to the dimensions of the term vectors, was configured during the clustering stage of the BoF algorithm. We investigated the parameter sensitivity of the automated classification test by varying these parameter values. In order for all images in a set to be tested, we employed the strategy of running several consecutive implementations of the BoF algorithm using the *k*-fold cross validation method with *k* = 4, and the performance results were represented as averaged values over the implementations. The numbers of images used for training and test samples are summarized in Figure S11.

## Data Availability Statement

The raw data supporting the conclusions of this manuscript will be made available by the authors, without under reservation, to any qualified researcher.

## Author Contributions

TS, SY, and TI designed the project. TS conducted the mouse model experiments and image acquisition. HN prepared the histological tissue sections. TW performed histological evaluation of the stained tissue slides. TS and ST implemented the digital image analysis. TS, ST, YH, and TI wrote the manuscript. All authors reviewed the manuscript.

### Conflict of Interest Statement

The authors declare that the research was conducted in the absence of any commercial or financial relationships that could be construed as a potential conflict of interest.
