# Supplementary material for: Tissue Intrinsic Fluorescence Spectra-Based Digital Pathology of Liver Fibrosis by Marker-Controlled Segmentation
Source: Front Med (Lausanne). 2018 Dec 11;5:350. doi: 10.3389/fmed.2018.00350 (PMC6297145; doi:10.3389/fmed.2018.00350)
Supplement: Supplementary file 1 [file Data_Sheet_1.PDF]

Supplementary Material

Tissue Intrinsic Fluorescence Spectra-Based Digital Pathology of Liver Fibrosis by Marker-Controlled Segmentation

Takashi Saitou*, Sota Takanezawa, Hiroko Ninomiya, Takao Watanabe, Shin Yamamoto, Yoichi Hiasa, Takeshi Imamura

*** Correspondence:** Takashi Saitou t-saitou@m.ehime-u.ac.jp

1 Supplementary methods

Immunohistochemistry

For preparation of cryosections, liver tissue samples preserved in 30% sucrose in PBS were embedded in optimal cutting temperature (OCT) compound (Sakura, Inc.). Then, the specimens were cut into 20 μm thick sections. Antigen retrieval was performed using 10 mM sodium citrate at 121°C for 3 min. After washing with tris-buffered saline with tween 20 (TBST), the samples were blocked with 1% goat serum in PBS before being subjected to antibody staining. The samples were incubated with α-SMA antibody (Cell Signaling Technology, Inc.) diluted in PBS supplemented with goat serum overnight at room temperature, followed by incubation with peroxidase-conjugated secondary antibody (Histofine simple stain MAX-PO(R), Nichirei Biosciences, Inc.) and detection was performed using the diaminobenzidine (DAB) system (Histofine simple stain DAB solution, Nichirei Biosciences, Inc.). For negative control samples, these same procedures were performed except for the α-SMA antibody incubation. Bright field images of the sections were acquired using a wide field inverted microscope (All-in-one fluorescence microscope BZ-X700, Keyence, Inc.) with a 20× magnification objective lens (PlanFluor 20× NA:0.45, Nikon).

2 Supplementary Figures

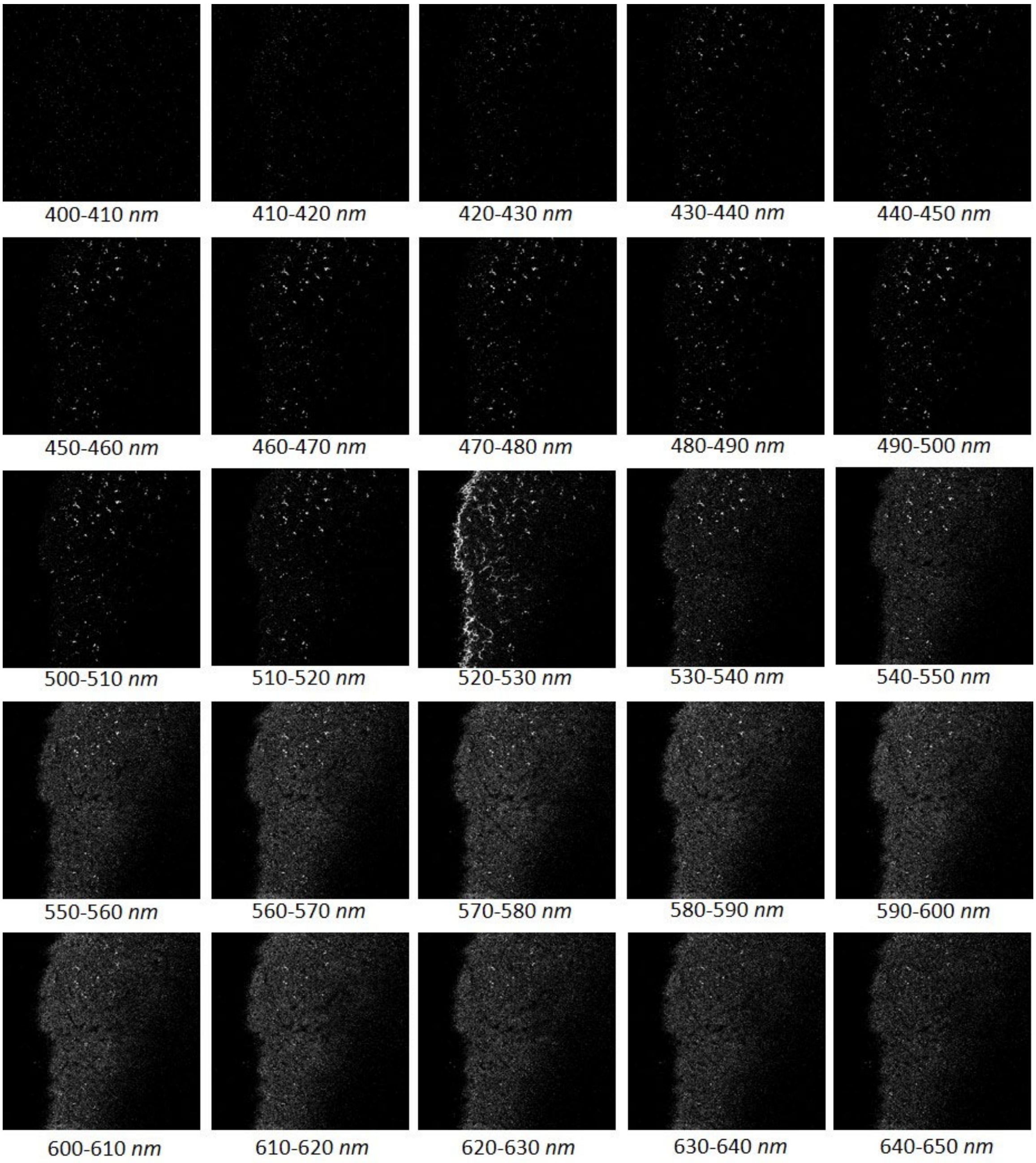

Figure S1. MP excitation spectral images for each spectral band. MP excitation image was acquired through the SD unit with an excitation wavelength 1050 nm. The emission spectra were detected as 25 channel images at wavelength range of 400–650 nm with bandwidth of 10 nm.

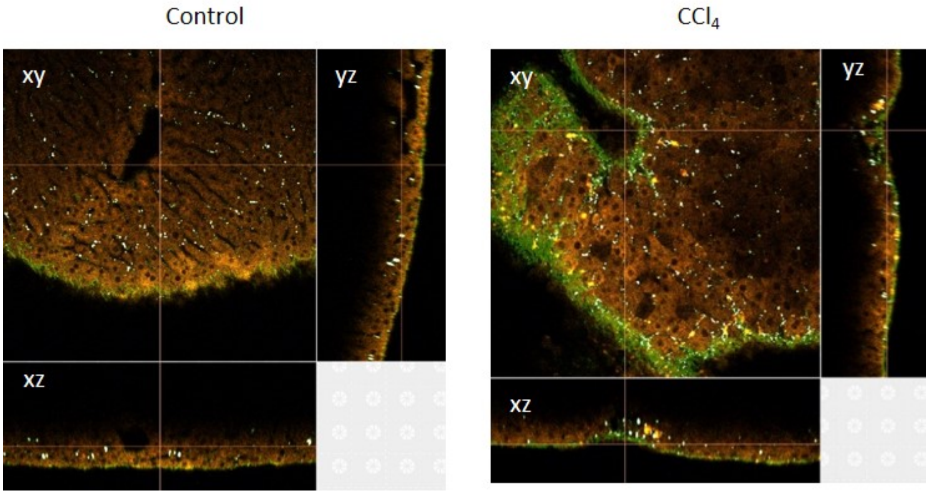

Figure S2. Slice views of MP excitation spectral images for liver tissues of the 2-week control and CCl_4 models.

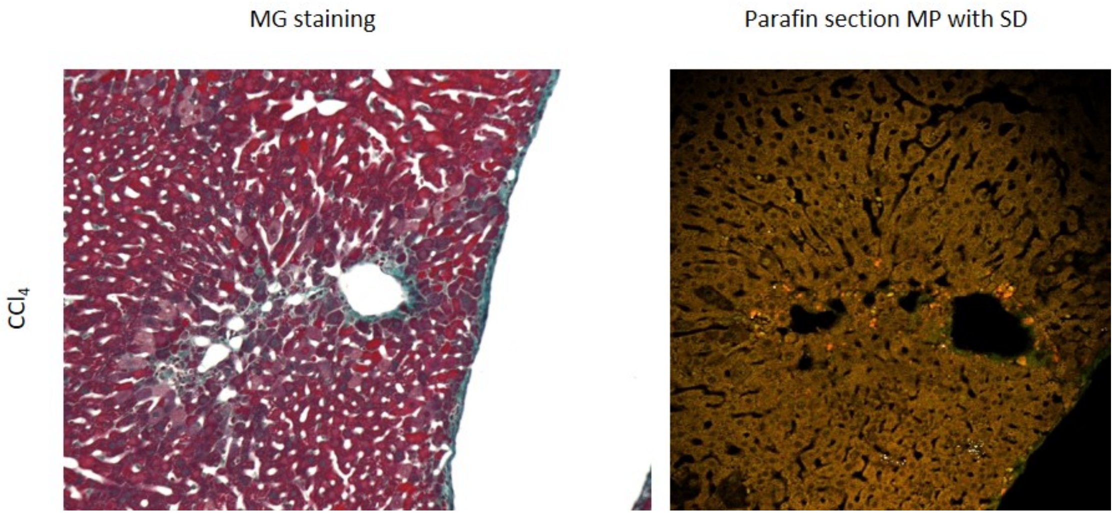

Figure S3. Comparison of the serial sections of histological and MP microscopy images from paraffin-embedded samples.

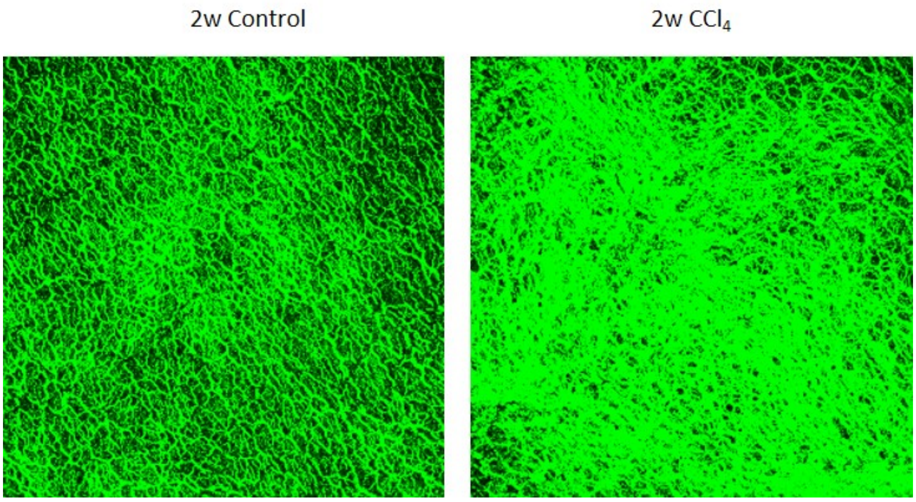

Figure S4. MIP images of the SHG signal for the 2-week control and CCl_4 models.

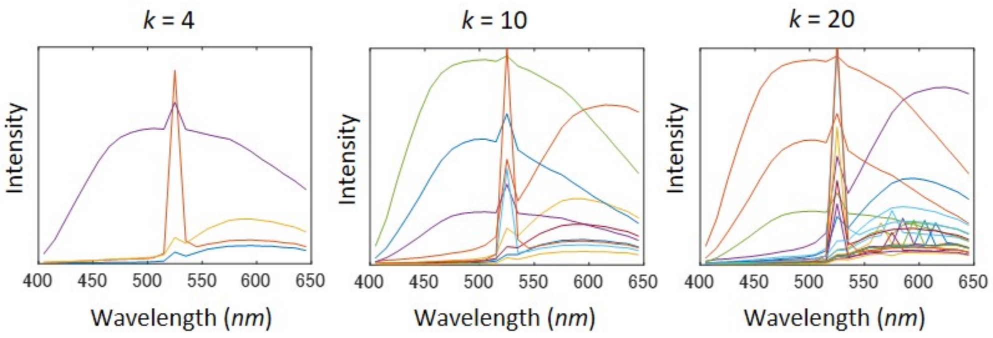

Figure S5. Quantization of the pixel-by-pixel collected spectra for different cluster numbers (*k* = 4, 10, 20).

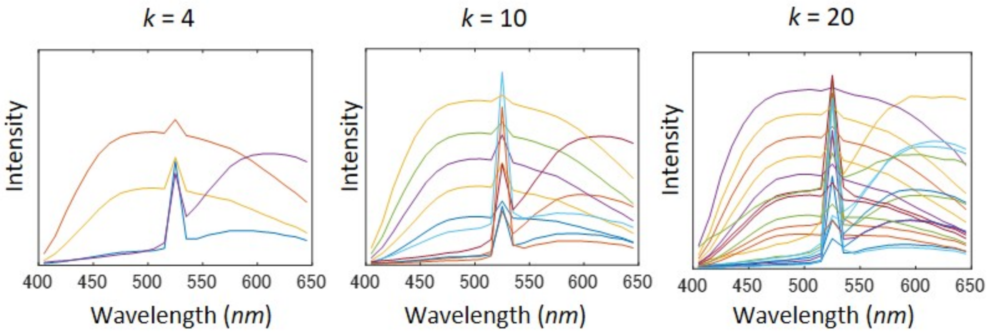

Figure S6. Quantization of the FAST feature-collected spectra for different cluster numbers (*k* = 4, 10, 20).

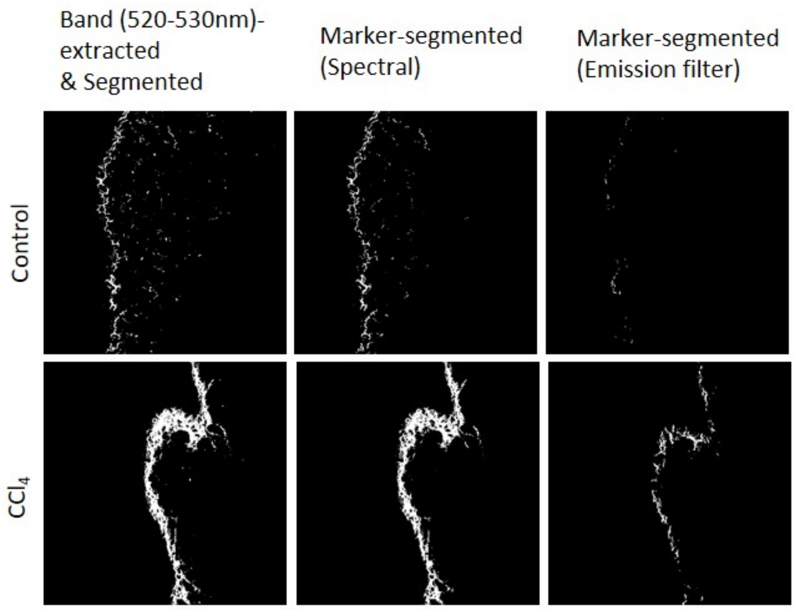

	Match rate (spectral)	Match rate (Emission-filter)
Control	0.9896	0.9266
CCl_4	0.9829	0.8635
Total	0.9859	0.8911

Figure S7. Comparison of the image segmentation results of the SHG signal. The segmentation images of the SHG band (520–530 nm) signal and marker-controlled segmentation images of the SHG signal for the spectral and three-channel image data through the emission filter sets are shown. The table represents the match rates between the spectral-band segmentation images and the marker-controlled segmentation image sets.

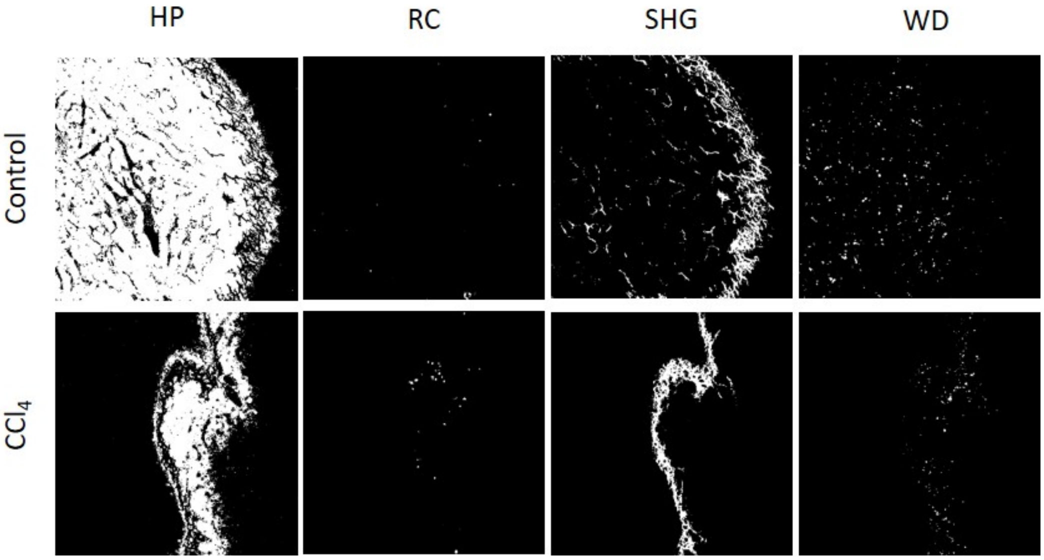

Figure S8. Marker-controlled segmentation images for HP, RC, SHG, and WD from the spectral image data. Marker-controlled segmentation images corresponding to the regions of HP, RC, SHG, and WD for the 2-week control and CCl_4 models are shown.

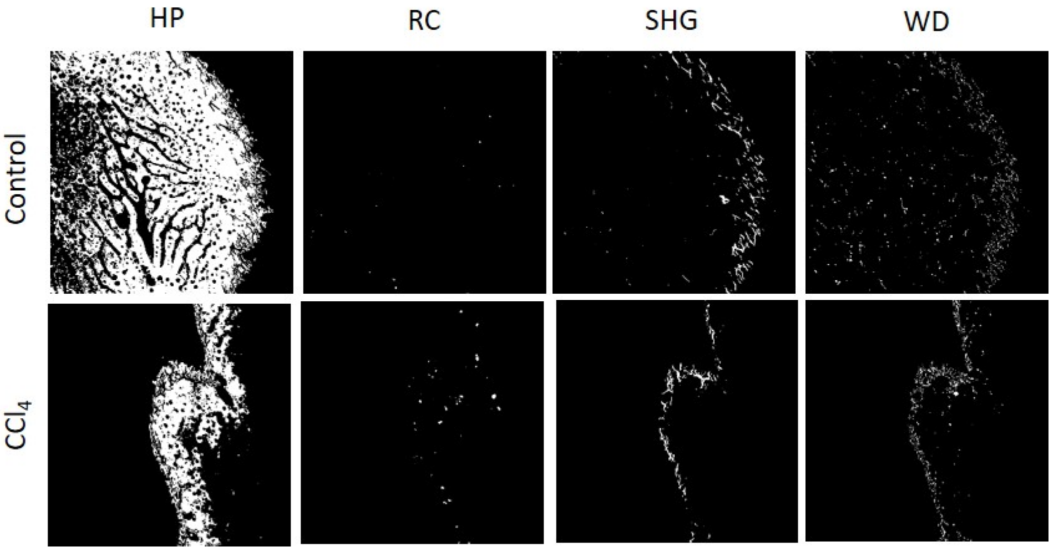

Figure S9. Marker-controlled segmentation images for HP, RC, SHG, and WD from the emission filter-based image data. Marker-controlled segmentation images corresponding to the regions of HP, RC, SHG, and WD for the 2-week control and CCl_4 models are shown.

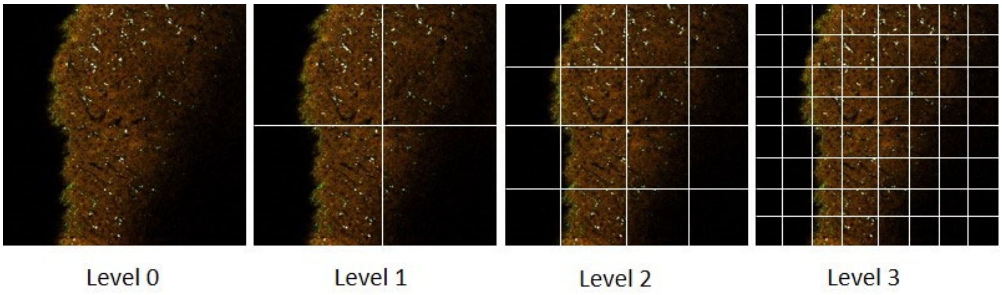

Figure S10. Definition of the image division level.

2-weeks control and CCl_4 classification	#Images for training	#Images for test
1	457	174
2	482	149
3	487	144
4	467	164

2-weeks CCl_4 and 4-weeks CCl_4 classification	#Images for training	#Images for test
1	694	219
2	668	245
3	653	260
4	724	189

Figure S11. Summary of the numbers of images used for training and test samples.

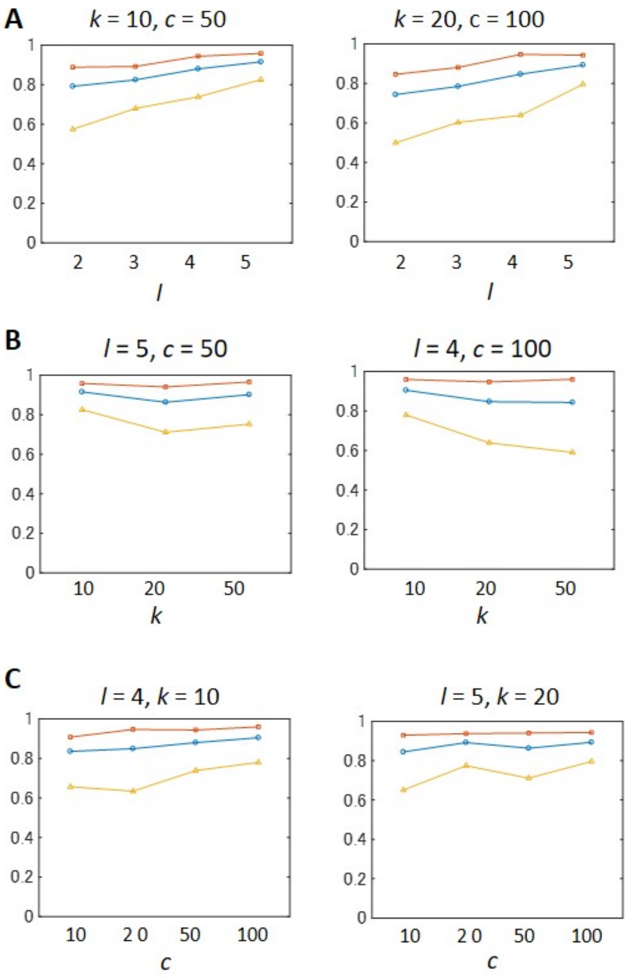

Figure S12. Results of the classification test performed on the 2-weeks models with various parameter values. The machine learning parameters are the division level *l*, the spectra cluster number *k*, and the codebook size *c*. The total, CCl_4, and control accuracy rates are depicted as blue, red, and yellow lines, respectively.

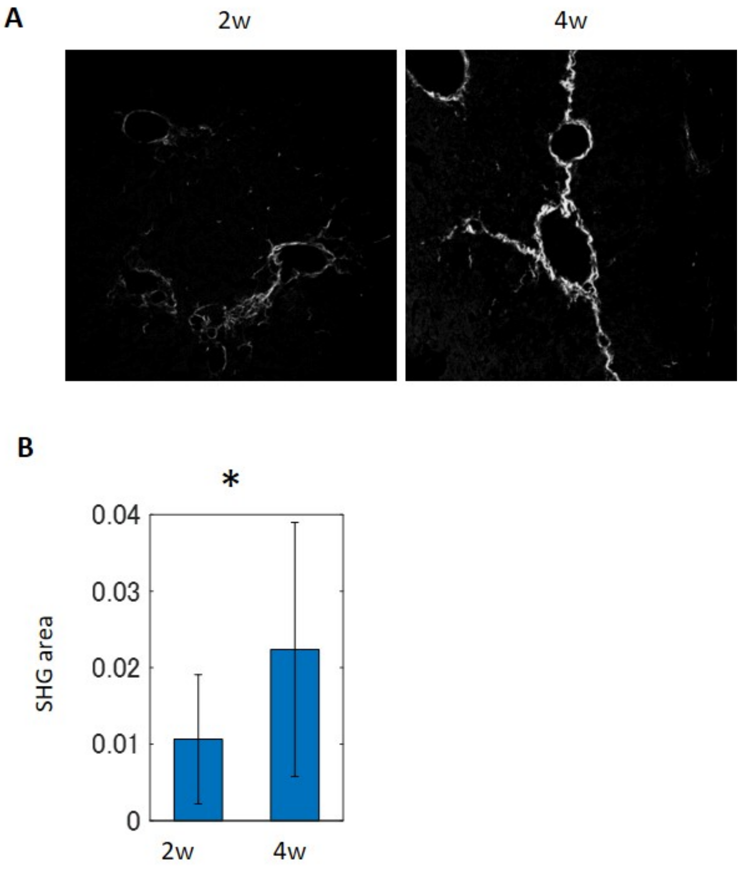

Figure S13. SHG images and quantification result of SHG area for tissue sections. (A) Representative SHG images for 2-week and 4-week CCl_4-induced fibrosis models. (B) The ratio of the SHG area to the total image area for the 2-week and 4-week CCl_4-induced fibrosis models. Asterisk indicates statistical significance with the Kolmogorov-Smirnov test with a p-value < 0.05.

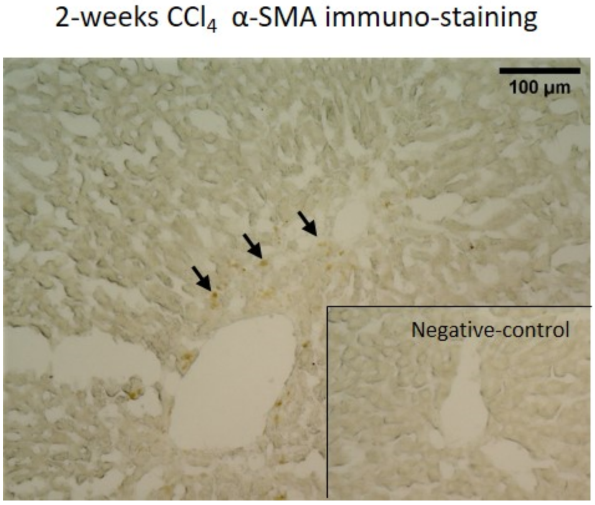

Figure S14. Immunohistochemical staining with α-SMA for the 2-week CCl_4 model liver tissues.

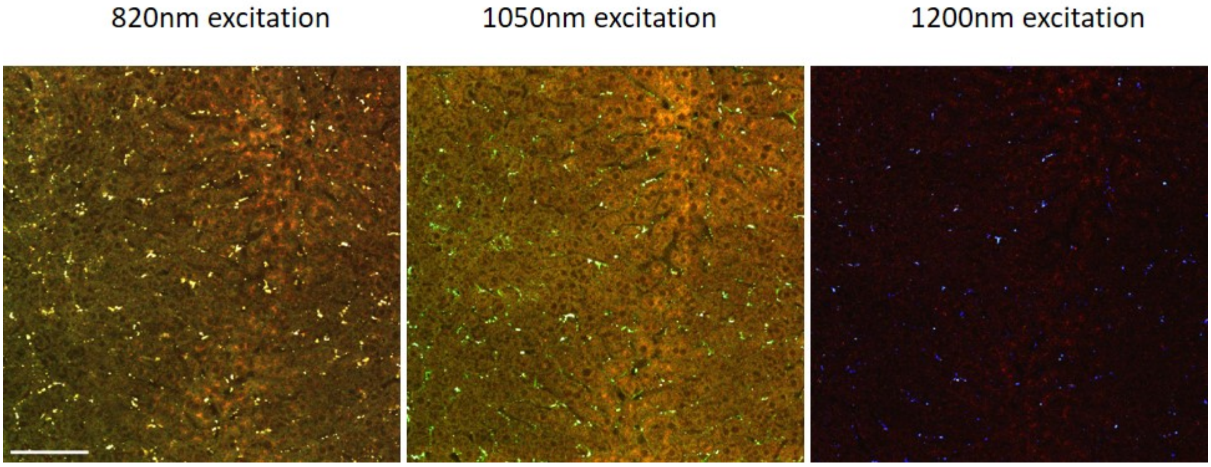

Figure S15. MP excitation images with different excitation IR laser wavelength. Images were acquired through the NDD unit with excitation wavelengths 800 nm, 1050 nm, and 1200 nm. Emission light was detected as four channel images through the bandpass filter sets, 417/60 nm (center wavelength/bandwidth), 525/50 nm, and 617/73 nm. Scale bar: 100 µm.
